# Supplementary material for: Insulin resistance markers HOMA-IR, TyG and TyG-BMI index in relation to heart failure risk: NHANES 2011-2016
Source: PLoS One. 2025 Sep 29;20(9):e0331740. doi: 10.1371/journal.pone.0331740 (PMC12478908; doi:10.1371/journal.pone.0331740)
Supplement: Attachment — Submitted filename: Response to Reviewers.docx [file pone.0331740.s006.docx]

**Response to the Reviewer’s Comments**

**Manuscript number:** PONE-D-24-43380

**Title:** Insulin Resistance Markers HOMA-IR and TyG Index in Relation to Heart Failure Risk: NHANES 2011-2016

**New Title:**Insulin Resistance Markers HOMA-IR, TyG and TyG-BMI Index in Relation to Heart Failure Risk: NHANES 2011-2016

**Reviewer #1:**

**Comment 1:** The description of heart failure is based on the patient's statement and is subjective. Of course, with this study method, there is no scientific and more accurate information based on clinical findings or echocardiography.

**Response 1:**

We sincerely appreciate the valuable comments from the reviewer. NHANES assesses heart failure using MCQ160B, a respondent-based self-report method that is common in large-scale epidemiological studies and has been widely used in epidemiological surveys. We fully understand the reviewer's concerns about the accuracy of self-reported diagnoses, and indeed, this method may introduce some recall bias. However, NHANES employs a standardized interview process, with data collection conducted by trained interviewers, which helps ensure the reliability of the data to some extent. Furthermore, we have added a note on the limitations of this method in the limitations section of the study and suggest that future research incorporate clinical data (such as echocardiograms or medical records) to further validate these findings. We thank the reviewer again for this suggestion, which helps us consider the limitations of the research methodology more comprehensively.

**Comment 2:** 13.72 percent of the patients had diabetes and it is not known what kind of treatment they received in order to calculate HOMA-IR.

**Response 2:**

We appreciate the reviewer’s valuable comments. We agree that diabetes treatment, especially insulin therapy, could affect the calculation of HOMA-IR, TyG, and TyG-BMI. Therefore, we conducted a subgroup analysis by grouping participants based on whether they had diabetes. We applied the strictest possible diagnostic criteria for diabetes and took into account the potential bias from self-reporting, further examining the use of antidiabetic medications and insulin, and incorporating laboratory indicators such as hemoglobin A1c to improve the diagnostic accuracy of diabetes. The subgroup analysis showed that the predictive ability of HOMA-IR and TyG for heart failure was indeed influenced in individuals with diabetes, which further supports the professionalism of the reviewer’s suggestion. Therefore, we included TyG-BMI to further refine the prediction, and the results indicated that TyG-BMI still demonstrated strong predictive ability in diabetic patients (OR = 1.003, P < 0.05). Additionally, we have added a note on the potential impact of diabetes treatment on the calculation of HOMA-IR in the limitations section of the study.

**Comment 3:** There is no indication of what type of treatment the patients received, perhaps for heart failure.

**Response 3:**

We appreciate the reviewer’s suggestion. We understand that heart failure treatment could potentially affect patients' metabolic status, thereby influencing the association between HOMA-IR and TyG indices. However, the NHANES dataset does not provide detailed information on heart failure treatment, so we were unable to directly adjust for this factor in our analysis. Nevertheless, the primary objective of this study was to assess the association between HOMA-IR, TyG indices, and heart failure, rather than to investigate the impact of treatment on this relationship. Additionally, we have acknowledged this limitation in the limitations section of our study and suggest that future research incorporate heart failure treatment data to further validate our findings.

**Revised text:**

(2) Self-reported bias: NHANES data relies on self-reports, especially for disease diagnoses and lifestyle-related data. This could introduce information bias, as self-reported data may not be entirely accurate or may be subject to recall bias. (3) Potential impact of diabetes on insulin resistance indices: Diabetes, especially in patients receiving insulin treatment, may affect the calculation of HOMA-IR and TyG indices. In this study, insulin treatment in diabetic patients may alter their metabolic characteristics, influencing the accuracy of insulin resistance indices. Although we considered diabetes as a factor in the analysis, the diversity of the diabetic population, including differences in diabetes type, control status, and medication use, was not fully controlled, which could lead to different metabolic characteristics in this group compared to non-diabetic patients. (4)Lack of HF treatment information: This study did not collect treatment information for HF patients, including medication use, surgical interventions, and other clinical management measures. HF treatment significantly impacts patient prognosis, so the lack of consideration of treatment interventions may affect the assessment of HF risk. Future studies should incorporate clinical data to further validate the findings of this study.

**Reviewer #2:**

**Comment 1:** Clarification of the non-linear relationship in HOMA-IR findings

A possible non-linear relationship between HOMA-IR and heart failure risk is mentioned. A more detailed explanation of this phenomenon, supported by additional data or references, would improve the understanding of the observed pattern.

**Response 1:**

We appreciate the reviewer’s suggestion. In our analysis, we observed a potential nonlinear relationship between HOMA-IR and heart failure risk, so we used restricted cubic splines (RCS) to further explore this relationship. The analysis showed that the association between HOMA-IR and heart failure risk was weak at lower levels, while the risk increased rapidly at higher levels, suggesting a possible threshold effect. Additionally, we have included an explanation of this phenomenon in the discussion section and suggest that future research further investigate the underlying biological mechanisms.

**Revised text:**

Notably, we found that the relationship between HOMA-IR and HF risk may not be linear. Some studies suggest that increasing insulin resistance could lead to cardiac metabolic disturbances and impaired cardiac function, but this process may not increase proportionally. The effect could intensify as the severity of insulin resistance increases. Mild insulin resistance may mainly affect metabolic health with minimal direct impact on the heart, whereas severe insulin resistance may significantly exacerbate cardiac load through inflammatory responses, fatty acid metabolism imbalance, and oxidative stress, thus increasing the risk of heart failure. Therefore, the relationship between insulin resistance and HF risk may not be simple linear, but rather exhibit varying effects depending on the degree of insulin resistance.

**Comment 2:** Consideration of external validity

Although the use of the NHANES database provides a solid foundation, discussing its applicability to other populations (e.g., different healthcare systems or ethnic groups underrepresented in NHANES) would strengthen the study’s relevance.

**Response 2:**

We appreciate the reviewer’s suggestion. As NHANES is a nationwide survey based on the U.S. population, its findings have a certain level of generalizability. However, we also acknowledge that this study is primarily based on the U.S. healthcare environment, and healthcare systems, racial compositions, and management strategies for metabolic diseases in different countries and regions may influence the relationship between HOMA-IR and heart failure risk. Therefore, we have included a discussion on external applicability in the discussion section and suggest that future research validate these findings in other populations (e.g., Asian and European populations) to further enhance the generalizability of the results.

**Revised text:**

(1)Population applicability: The study used data from the NHANES database. Although this dataset offers broad population representation, its specific characteristics of the U.S. population may affect the generalizability of the results. Specifically, since NHANES data is mainly from the U.S., it may not fully represent populations from other countries or regions.

**Comment 3:** Greater emphasis on causal relationships

It is acknowledged that the study is observational in nature, but further exploration of possible causal mechanisms or references to longitudinal studies could enhance the interpretation of the results

**Response 3:**

We appreciate the reviewer’s suggestion. As this study is based on cross-sectional data, we cannot directly infer the causal relationship between HOMA-IR, TyG index, and heart failure. We have found that some prospective cohort studies also support TyG as a potential causal risk factor for heart failure. We have added this point to the discussion section and suggest that future studies adopt longitudinal research or causal inference methods to further validate the potential causal effects of HOMA-IR, TyG, and TyG-BMI indices on heart failure risk.

**Revised text:**

A prospective cohort study in an Asian population examining the long-term relationship between TyG and HF risk showed that after adjusting for potential confounders, Cox regression analysis revealed hazard ratios of 1.02, 1.29, and 1.40 for Q2, Q3, and Q4 groups, respectively. Subgroup analyses also showed a significant interaction between cumulative TyG index and BMI or waist circumference. These findings are consistent with our results.

**Reviewer #3:**

**1) Paper formatting issues**

**Comment 1:** This manuscript is not in the correct format for PLOSONE.

**Response 1:**

We appreciate the reviewer’s comment. We have carefully reviewed the "Author Guidelines" of PLOS ONE and made comprehensive adjustments to the manuscript format to ensure it complies with the journal's requirements. These modifications include adjusting the title format, reference style, and table presentation. We believe these changes will enhance the readability of the paper and align it with the submission guidelines of PLOS ONE.

**Comment 2:** Moreover, even though, the writing is sufficient, I am very annoyed with the improper use of abbreviation. Please follow the “Instruction to the authors”, specifically for abbreviation.

**Response 2:**

We appreciate the reviewer’s feedback and have thoroughly checked the use of abbreviations in the manuscript. In accordance with PLOS ONE's requirements, we have made the necessary adjustments, such as providing the full spelling of terms at first use, removing unnecessary abbreviations, and standardizing the use of abbreviations throughout the text to ensure consistency and readability. We believe these adjustments will enhance the readability of the paper and align it with PLOS ONE's writing guidelines.

**Comment 3:** Also, when reporting p-values, always used 3 significant figures.

**Response 3:**

We appreciate the reviewer’s thorough review and have made the necessary adjustments in accordance with PLOS ONE's requirements, standardizing the reporting of p-values to three significant digits. All p-value modifications have been updated in the manuscript and are consistent throughout the tables and main text.

**2) Abstract section**

**Comment 1:** In the abstract, please add some results to support your conclusion.

**Response 1:** We appreciate the reviewer’s suggestion and have added more research results to the abstract to better support the conclusions. Specifically, we have included the specific associations between HOMA-IR, TyG, and TyG-BMI with heart failure risk, and clearly reported the results of the ROC curve analysis in the abstract. We believe these additions will better support the research conclusions and improve the quality of the abstract’s expression.

**Comment 2:** Also, Line 34: I am not sure if “TyG index outperforms HOMA-IR in predicting HF risk”. Please show the comparison results.

**Response 2:** We appreciate the reviewer’s suggestion and have provided more direct comparative data in the statement on line 34.

**Revised text:**

Results: HOMA-IR (OR = 1.017, 95% CI: 1.006-1.027, P < 0.01), TyG (OR = 1.798, 95% CI: 1.453-2.225, P < 0.001), and TyG-BMI (OR = 1.006, 95% CI: 1.004-1.008, P < 0.001) were all significantly associated with HF risk, with TyG showing the strongest association. ROC curve analysis demonstrated that TyG (AUC = 0.61) and TyG-BMI (AUC = 0.62) had better predictive abilities than HOMA-IR (AUC = 0.6). In subgroup analyses, HOMA-IR showed higher sensitivity in the female population, while TyG-BMI provided a complementary role to TyG in individuals with diabetes.

**3) Concerning the methods**

**Comment 1:** Why were only 2011-2016 cycles were used? MCQ160B is reported for all cycles.

**Response 1:**

We appreciate the reviewer’s attention to our study and the suggestions provided. The main reasons for selecting the NHANES 2011-2016 data cycle are as follows:

(1) **Data Completeness and Availability:** NHANES is an ongoing cross-sectional survey, but the variables collected may differ across years. Although the MCQ160B (heart failure) variable is reported in multiple cycles, we found that the NHANES data from 2011-2016 had the most complete and high-quality data for calculating HOMA-IR and TyG indices (such as fasting glucose, fasting insulin, triglycerides, etc.). Therefore, to ensure the integrity and consistency of the study data, we selected the 2011-2016 data cycle.
(2) **Methodological Consistency:** NHANES may adjust laboratory testing methods, questionnaire surveys, and other processes across different cycles. To avoid potential systematic errors, we chose the relatively newer 2011-2016 data to ensure the stability and comparability of the study results.
(3) **Data Representativeness:** Although extending the study period may increase the sample size, NHANES uses a complex multi-stage sampling design, and the data from each two-year cycle already provides a representative sample of the U.S. population. Based on the 2011-2016 data, we were still able to obtain a representative study sample, ensuring statistical power.

**Comment 2:** Also, considered also examining coronary heart disease (MCQ160C) and heart attack (MCQ160E).

**Response 2:**

We greatly appreciate the reviewer’s suggestion and agree that there may be some connections between different subtypes of cardiovascular diseases. However, the core objective of this study is to explore the relationship between insulin resistance and heart failure, while coronary artery disease and myocardial infarction differ from heart failure in terms of pathophysiological mechanisms and clinical presentation. Given the main hypothesis and the research design, we chose to focus on heart failure to avoid the inclusion of excessive disease endpoints that could impact the specificity of the study. Additionally, the cross-sectional design of NHANES limits causal inference, and including other cardiovascular diseases might lead to interpretational confounding. Therefore, we believe the current scope of the study is appropriate.

**Comment 3:** Please explain why estimated glomerular filtration rate (eGFR) was used as an exclusion criterion?

**Response 3:** We appreciate the reviewer’s suggestion. Since the focus of this study is to explore the association between HOMA-IR, TyG, and TyG-BMI with heart failure, we aimed to minimize confounding effects caused by chronic kidney disease. At the same time, we sought to avoid bias due to end-stage renal disease to ensure the stability of the study results.

**Comment 4:** Were certain medications also considered?

**Response 4:** We appreciate the reviewer’s suggestion. This study did not adjust for the use of all medications, but we acknowledge that certain drugs (such as antidiabetic medications, diuretics, β-blockers, etc.) may affect HOMA-IR, TyG, TyG-BMI. Therefore, we have added a discussion in the limitations section regarding the potential impact of medications and suggest that future studies further investigate the interference of drug treatments on this association.

**Revised text:**

(3) Potential impact of diabetes on insulin resistance indices: Diabetes, especially in patients receiving insulin treatment, may affect the calculation of HOMA-IR and TyG indices. In this study, insulin treatment in diabetic patients may alter their metabolic characteristics, influencing the accuracy of insulin resistance indices. Although we considered diabetes as a factor in the analysis, the diversity of the diabetic population, including differences in diabetes type, control status, and medication use, was not fully controlled, which could lead to different metabolic characteristics in this group compared to non-diabetic patients. (4)Lack of HF treatment information: This study did not collect treatment information for HF patients, including medication use, surgical interventions, and other clinical management measures. HF treatment significantly impacts patient prognosis, so the lack of consideration of treatment interventions may affect the assessment of HF risk. Future studies should incorporate clinical data to further validate the findings of this study.

**Comment 5:** Please consider examine the effect the TyG-BMI index has on Heart Failure. The TyG-BMI is considered superior to the TyG index.

**Response 5:**

We appreciate the reviewer’s suggestion. TyG-BMI is a novel composite index related to insulin resistance, which has shown predictive ability for heart failure (HF) in some studies. To further enhance the analysis, we calculated the TyG-BMI index using the NHANES 2011-2016 data as suggested by the reviewer and evaluated its association with HF risk.

The results showed that the relationship between TyG-BMI and HF risk was similar to that of TyG and remained significant after adjusting for covariates. In the unadjusted model, TyG-BMI was significantly associated with HF (OR = 1.006, 95% CI = 1.004-1.008, P < 0.01), and in the Q4 group, for each 1-unit increase in TyG-BMI, the risk of HF increased by 2.6 times. TyG-BMI exhibited stable predictive ability across models 1-3. Additionally, when comparing the predictive abilities of TyG, TyG-BMI, and HOMA-IR, we found that the AUC for TyG-BMI was slightly higher than for TyG, but the difference was not statistically significant. Further subgroup analyses revealed that TyG-BMI had predictive ability in individuals with diabetes, which may be an important supplement to TyG.

We have added the calculation method for TyG-BMI in the methods section and included the relevant analysis in the results section. In the discussion section, we have also added the clinical significance of the TyG-BMI index and a comparison with TyG. We sincerely thank the reviewer for the valuable suggestions, which have improved our study.

**Revised text:**

In addition, the TyG-BMI is calculated by combining the TyG index with BMI, using the formula: TyG-BMI = TyG × BMI. TyG-BMI has been proposed as a novel marker for assessing IR, as it can integrate the influence of body weight factors on IR.

Based on the distribution of HOMA-IR, TyG, and TyG-BMI values, participants were further categorized into four quartiles: Q1, Q2, Q3, and Q4, representing different ranges of HOMA-IR, TyG, or TyG-BMI values. Specifically, Q1 corresponds to the lowest quartile (HOMA-IR < X or TyG < A or TyG-BMI < B), while Q4 represents the highest quartile (HOMA-IR ≥ Y or TyG ≥ C or TyG-BMI ≥ D). These quartile groupings were used to explore the relationship between HOMA-IR, TyG, or TyG-BMI and the study outcomes.

**Comment 6:** Would the authors consider using HbA1c for T2D and blood pressure for hypertension to confirm the presence of these pathologies? As well as potentially examine the RXQ files to see which medications the participants are using.

**Response 6:**

We appreciate the reviewer’s insightful comment regarding the diagnosis of diabetes and hypertension. Indeed, relying solely on self-reported data to diagnose diabetes (DIQ010) or hypertension (BPQ020) may introduce bias. In response to the reviewer’s suggestion, we incorporated HbA1c (LBXHG) and used a cutoff of HbA1c ≥ 6.5% to further diagnose diabetes. Additionally, we included data from three separate blood pressure measurements (systolic BP: BPXSY1-BPXSY1, diastolic BP: BPXDI1-BPXDI3) and calculated the average of these three measurements. Based on SBP ≥ 130 mmHg or DBP ≥ 80 mmHg, we further diagnosed hypertension.

We fully agree with the reviewer’s point on addressing medication use to reduce the potential for missed diagnoses. Following the reviewer’s suggestion, we reviewed the RXQ file to extract medication information. However, the RXQ file in the NHANES database removed descriptions of medication categories after 2002, and the list of individual drug names in the RXQ file made it challenging to extract the relevant medication categories. Nevertheless, we focused on the use of insulin and anti-diabetic medications in diabetic patients, as well as antihypertensive medications in hypertensive patients. Therefore, we included DIQ050, DIQ070, BPQ040A, and BPQ050A as alternative measures and further confirmed the diagnosis of diabetes and hypertension based on medication use.

**Revised text:**

At the same time, we included diabetes and hypertension as covariates in the analysis. For diabetes diagnosis, we combined self-reported data from participants (DIQ010) with the use of insulin (DIQ050) and antidiabetic medications (DIQ070), and included glycated hemoglobin (LBXGH) to improve the detection rate of diabetes. Diabetes was diagnosed when glycated hemoglobin ≥ 6.5%. For hypertension diagnosis, we combined self-reported data (BPQ020) with the use of antihypertensive medications (BPQ040A, BPQ050A), and calculated the average of three blood pressure measurements (BPXSY1-BPXSY3, BPXDI1-BPXDI3) to improve the diagnosis rate of hypertension. Hypertension was diagnosed when systolic blood pressure ≥ 130 or diastolic blood pressure ≥ 90. The selection of these covariates was based on their potential impact on metabolic health, cardiovascular disease risk, and other chronic conditions.

**Comment 7:** Please indicate which R packages were used and for which analysis.

**Response 7:** We appreciate the reviewer’s valuable suggestion. We have added the R packages used and their specific purposes in the revised manuscript.

**Revised text:**

The main R packages used in this study included: dplyr (version 1.1.4) for data manipulation and cleaning, ggplot2(version 3.5.1) for data visualization, mice (version 3.16.0) for multiple imputation of missing data, rms (version 6.8-2) for constructing nomogram models, pROC (version 1.18.5) for ROC curve analysis, and car (version 3.1-3) for multivariable regression analysis. These R packages and versions ensure the transparency and reproducibility of the analysis.

**Comment 8:** Lines 140-142: please provide a description of which test were used.

**Response 8:** We appreciate the reviewer’s valuable suggestion. We have specifically described the statistical testing methods in the revised manuscript.

**Revised text:**

Stratified analyses were conducted based on age, sex, race/ethnicity, diabetes status, and hypertension. Interaction P-values were calculated using likelihood ratio tests in multivariable logistic regression (LRT) models to assess the consistency of relationships across subgroups. A predictive nomogram model incorporating key HF-related variables was developed using multivariable logistic regression, and its discriminative ability was evaluated using receiver operating characteristic (ROC) curves with the area under the curve (AUC) calculation and decision curve analysis (DCA) to assess clinical utility.

**Comment 9:** Were the sample weights corrected for the number of cycles used? Supplement 1 does not suggest they were.

**Response 9:**

We appreciate your valuable suggestion. We acknowledge that the sample weights were not previously adjusted for the number of cycles. To ensure the accurate representativeness of the NHANES 2011-2016 data, we have corrected the 2-year weight (WTMEC2YR) by dividing it by 3, and the new weight is denoted as WTMEC6YR. Please refer to Supplement 1 for details, and all statistical analyses have been updated accordingly.

**Revised text:**

To ensure the accurate representativeness of the NHANES 2011-2016 data, we have adjusted the 2-year weight (WTMEC2YR) by dividing it by 3, as per the NHANES guidelines (WTMEC6YR).

**Comment 10:** Lastly, please indicate, by model, which variables were used. Do not use “and others to account for potential confounders”.

**Response 10:** We appreciate the reviewer’s suggestion. We have revised the original vague expression and specifically outlined the variables adjusted for in the five models.

**Revised text:**

Model I adjusted for sex, age, and race/ethnicity. Model II further adjusted for hypertension, total cholesterol, low-density lipoprotein cholesterol, and serum sodium levels. Model III additionally adjusted for diabetes. Model IV further incorporated adjustments for serum potassium, serum uric acid, alanine aminotransferase (ALT), aspartate aminotransferase (AST), creatinine, and albumin. Model V included all covariates.

**4) Results section**

**Comment 1:** I do not like the phrasing here, Lines 155-157. Also, the number of participants should not be included because the percentage is based on weighted values.

**Response 1:**

We appreciate the reviewer’s suggestion. Indeed, since NHANES employs weighted analysis, our results should be based on the weighted estimates rather than the raw sample size.

**Revised text:**

The average age of the study population was 48.25 years. Males accounted for 48.60% of the weighted sample, and non-Hispanic Whites constituted the largest racial group, also comprising 48.60%. The weighted prevalence of heart failure was 3.70%.

**Comment 2:** Lines 165-166: Repetitive information. Also, I think it would be better to combine Tables 1 and 2.

**Response 2:**

We appreciate the reviewer’s suggestion. We have combined Table 1 and Table 2, updated the statistical information for hypertension and diabetes, and added the statistical results for TyG-BMI.

**Comment 3:** Check the units in Table 1, specifically, for TC, LDL, and HDL.

**Response 3:**

We appreciate the reviewer for pointing out our mistake, and we sincerely apologize for the error in the unit information. We are grateful for the reviewer's attention to detail and thoroughness. We have carefully reviewed and corrected the units for each indicator.

**Comment 4:** Lines 202-205: Where is the data to support this claim, specifically for the “sensitivity analysis”. I think the term maybe wrong?

**Response 4:**

We appreciate the reviewer for pointing out our mistake. It seems there was a language and data error, and we have removed that part of the content.

**Comment 5:** Lines 217-220: I cannot agree this statement, “indicating greater sensitivity of HOMA-IR to heart failure risk among men”. The authors need to test if there is a significant difference between the 2 ORs.

**Response 5:**

We appreciate the reviewer for pointing out our mistake. To examine the differential impact of HOMA-IR on heart failure risk in males and females, we included an interaction term between HOMA-IR and sex (RIAGENDR) in the model and conducted a significance test. The results showed that the effect of HOMA-IR on heart failure risk was significantly different in males (OR: 1.729, 95% CI: 1.001–1.015, P < 0.05) and females (OR: 1.905, 95% CI: 1.021–1.055, P < 0.001), with the interaction showing P < 0.05. Specifically, the interaction test revealed that the effect of HOMA-IR on heart failure risk was more pronounced in females.

**Revised text:**

For HOMA-IR, an increase was significantly associated with heart failure risk in both males (OR: 1.729, 95% CI: 1.001–1.015, P < 0.05) and females (OR: 1.905, 95% CI: 1.021–1.055, P < 0.001), with the interaction suggesting that it may be more sensitive in females (P < 0.05).

**Comment 6:** Lines 246-247: HOMA-IR does not impact heart failure, but insulin resistance. Moreover, this is discussion and should not be here.

**Response 6:** We appreciate the reviewer's suggestion, and we have removed this part of the content.

**Comment 7:** Lines 255-257: Please perform the AUC comparison analysis.

**Response 7:** We appreciate the reviewer’s suggestion. We have added the content for the AUC comparison analysis.

**Revised text:**

In this study, we systematically assessed the effectiveness of HOMA-IR, TyG, and TyG-BMI in predicting HF risk. Through ROC curve analysis, we found that the area under the curve (AUC) for TyG-BMI (AUC=0.62) was higher than that for TyG (AUC=0.61), which was higher than HOMA-IR (AUC=0.60), indicating the superiority of TyG-BMI and TyG in identifying HF risk (Figure 3). Additionally, decision curve analysis (DCA) further confirmed the clinical applicability of TyG and TyG-BMI across different decision thresholds, demonstrating their higher decision value (Figure 4). In the restricted cubic spline (RCS) curve analysis, both TyG (Figure 5) and TyG-BMI (Figure 6) exhibited a positive correlation trend with HF risk, while HOMA-IR did not demonstrate the same prognostic capability (Figure 7).

**Comment 8:** The figures are very blurry.

**Response 8:**

We appreciate the reviewer’s valuable feedback. We have increased the resolution of the image to improve its clarity. The revised high-resolution image has been uploaded and is available for download.

**5) Discussion section**

**Comment 1:** Lines 278-279: the authors mention that “HF is closely related to various risk factors, including hypertension, coronary artery disease,”; therefore, I believe this analysis could be included.

**Response 1:**

We appreciate the reviewer’s valuable feedback. Although we did not include hypertension and coronary artery disease variables in our analysis, we acknowledge that these conditions are indeed closely related to heart failure. We will cite relevant literature in the discussion section to emphasize the role of these factors in heart failure risk.

**Comment 2:** In the discussion, do not repeat the results. (Lines: 301-303; 305-306).

**Response 2:**

We appreciate the reviewer’s valuable feedback. We have removed the repetition of the results discussion. We will revise the discussion section to focus on interpreting the study findings, comparing them with existing literature, and highlighting the significance of the results, without repeating the results already presented.

**Comment 3:** Lines 312-314: the authors indicate that “previous studies” but do not reference any source. Please make sure all statements are properly cited.

**Response 3:** We appreciate the reviewer’s valuable feedback, and we have completed the relevant additions.

**Comment 4:** Please add more limitations. You have at least potential reporting bias.

**Response 4:**

We appreciate the reviewer’s valuable suggestions. We have added more limitations in the revised manuscript, including the applicability of the population, the potential reporting bias from self-reported data, the possible influence of diabetes on insulin resistance indices, the lack of information on heart failure treatment, and the limitations associated with the cross-sectional study design.

**Revised text:**

Despite the important insights provided by this study, there are some limitations. These include: (1) Population applicability: The study used data from the NHANES database. Although this dataset offers broad population representation, its specific characteristics of the U.S. population may affect the generalizability of the results. Specifically, since NHANES data is mainly from the U.S., it may not fully represent populations from other countries or regions. (2) Self-reported bias: NHANES data relies on self-reports, especially for disease diagnoses and lifestyle-related data. This could introduce information bias, as self-reported data may not be entirely accurate or may be subject to recall bias. (3) Potential impact of diabetes on insulin resistance indices: Diabetes, especially in patients receiving insulin treatment, may affect the calculation of HOMA-IR and TyG indices. In this study, insulin treatment in diabetic patients may alter their metabolic characteristics, influencing the accuracy of insulin resistance indices. Although we considered diabetes as a factor in the analysis, the diversity of the diabetic population, including differences in diabetes type, control status, and medication use, was not fully controlled, which could lead to different metabolic characteristics in this group compared to non-diabetic patients. (4)Lack of HF treatment information: This study did not collect treatment information for HF patients, including medication use, surgical interventions, and other clinical management measures. HF treatment significantly impacts patient prognosis, so the lack of consideration of treatment interventions may affect the assessment of HF risk. Future studies should incorporate clinical data to further validate the findings of this study. (5) Limitations of cross-sectional design: As a cross-sectional study, this research can only reveal the association between HOMA-IR, TyG, and TyG-BMI with HF, but cannot establish causality. Therefore, although we observed a strong correlation between these metabolic indices and HF risk, it does not imply that they are direct causes of HF occurrence.
