# Supplementary material for: Insulin resistance markers HOMA-IR, TyG and TyG-BMI index in relation to heart failure risk: NHANES 2011-2016
Source: PLoS One. 2025 Sep 29;20(9):e0331740. doi: 10.1371/journal.pone.0331740 (PMC12478908; doi:10.1371/journal.pone.0331740)
Supplement: Attachment — Submitted filename: Response_to_Reviewers_auresp_2.docx [file pone.0331740.s007.docx]

**Response to the Reviewer’s Comments**

**Manuscript number:** PONE-D-24-43380

**Title:** Insulin Resistance Markers HOMA-IR and TyG Index in Relation to Heart Failure Risk: NHANES 2011-2016

**New Title:**Insulin Resistance Markers HOMA-IR, TyG and TyG-BMI Index in Relation to Heart Failure Risk: NHANES 2011-2016

We would like to sincerely thank the editors and reviewers of ***PLOS ONE*** for dedicating their time and effort to review our manuscript and provide valuable feedback.

However, we have noticed that the version reviewed by the reviewers seems to be the initial submission before the first round of revisions, rather than the updated version we submitted after addressing the feedback. We have carefully integrated all the reviewers' comments from both rounds of review and have made detailed revisions in the updated manuscript.

We kindly ask the editors and reviewers to review the revised version so that they can more accurately assess the changes we have made. Thank you for your understanding, and we look forward to receiving your further feedback.

**Reviewer #3:**

1) **Format**

**Comment 1:**
a) I think this is not in the correct format for PLOS one. Please fix it.
b) The references although current, are not in a consistent format.
c) The figures are blurry. Figure 2 is unreadable. Please resolve this issue. Some of the numbers look the same for both TyG and HOMA-IR. Maybe a table. Figures 5 and 6 is Figure 7, I suggest using only Figure 7.
d) Use abbreviation consistently. If you abbreviate in the discussion, then it should have been used throughout the manuscript.

**Response 1:**

Thank you for your careful and detailed feedback regarding the formatting of our manuscript. We have made comprehensive revisions to the manuscript format according to the guidelines of PLOS ONE. The specific changes are as follows:

(1) We have restructured the entire manuscript to align with the official formatting guidelines of PLOS ONE, including title hierarchy, paragraph styles, line spacing, figure numbering, and citations, ensuring that the format complies with the journal’s requirements.
(2) We have standardized the format of all references, aligning them with the citation style of PLOS ONE. Additionally, we have used reference management tools to automatically ensure consistency.
(3) We carefully considered the reviewer’s suggestion regarding Figure 2. Given the large amount of data in Figure 2, and to enhance the readability for readers while considering the journal's page limits, we followed the reviewer’s recommendation and replaced Figure 2 with a table to improve data presentation. Regarding the suggestion that "Figures 5 and 6 duplicate the content of Figure 7," after a thorough review, we confirmed that Figures 5, 6, and 7 represent the nonlinear relationships between TyG, TyG-BMI, and HOMA-IR with heart failure risk, respectively. Since the indicators and implications are distinct, we believe each figure should be displayed independently. We have re-created the figures and clarified the legends and titles to avoid any confusion.
(4) We have thoroughly checked and standardized all abbreviations throughout the manuscript. Full terms are now provided at the first occurrence of each abbreviation, with consistent use of the abbreviation throughout the rest of the manuscript (e.g., heart failure is consistently abbreviated as HF, insulin resistance as IR, etc.), ensuring linguistic consistency and professionalism.

Once again, we appreciate the valuable suggestions provided by the reviewer, which have significantly contributed to improving the quality of our manuscript.

**2) Methodology**

**Comment 2:** Why were years 2011-2016 were used? Data for these indices as well as Heart Failure is available for 1999-2018. I can understand not using cycle P and L as for COVID, but I think maybe other cycles could be included.

**Response 2:**

Thank you for the important suggestion regarding the selection of the study period. During the study design phase, we conducted a preliminary review and comparison of data from multiple NHANES cycles and ultimately selected the period from 2011 to 2016 for the following reasons:

(1) Variable Consistency and Availability: HOMA-IR, TyG, and TyG-BMI require the joint calculation of several key variables (including insulin, fasting glucose, and triglycerides). However, some earlier NHANES cycles (e.g., 1999–2006) have missing data or inconsistent measurement methods for these variables, which could impact the accuracy and comparability of the analysis.
(2) Data Quality and Recency: The data from 2011 to 2016 have high data quality, lower missing rates, and a higher degree of standardization in laboratory testing methods, ensuring the stability of the model analysis and the reproducibility of results.

Thus, while data from other years may also be valuable for research, we believe that the 2011–2016 period best meets the objectives of this study and ensures the reliability and consistency of the analysis results. We will further explain the rationale for our data selection in the discussion section to address the reviewer’s concern.

**Comment 3:** You used the TyG index as a potential superior index to HOMA-IR; however, TyG-BMI is potentially superior to TyG. Please consider using this index as well.

**Response 3:**

Thank you for your valuable suggestion. We fully agree with the importance of TyG-BMI as an emerging insulin resistance index, which may have advantages in predicting heart failure risk. In this study, we have systematically compared TyG-BMI with TyG and HOMA-IR through the following analyses:

(1) In multivariable logistic regression, we compared the associations between each of the three indices and heart failure risk.
(2) We used ROC curves and AUC values to evaluate the predictive abilities of the three indices.

(3)We further compared the clinical utility and nonlinear trends of the three indices using decision curve analysis (DCA) and restricted cubic splines (RCS).

(4)We also showcased the predictive performance of TyG-BMI in subgroup analyses.

Additionally, in the discussion section, we specifically highlighted the potential value of TyG-BMI in enhancing the predictive ability of TyG in diabetic populations.We appreciate the reviewer’s important suggestion, which has helped make our research more complete and rigorous.

**Comment 4:** You used quartiles to analyze the risk; however, how many participants jumped quartile between the 2 indices.

**Response 4:**

Thank you for your feedback. After further analysis of the data, we found that although we used quartiles for grouping, there was minimal variation in the quartile distribution between TyG, HOMA-IR, and TyG-BMI. As a result, we did not observe significant changes in the quartiles. Specifically, the distribution of all quartiles was relatively consistent, and participants remained in the same quartile across different indices. We have examined the quartile distributions for each variable and found that all data points were evenly distributed across the quartiles, with no significant cross-quartile shifts.

We will continue to explore the potential characteristics of the data to ensure the accuracy of the analysis results. Once again, we appreciate the valuable feedback provided by the reviewer, and we have updated the discussion section in the revised manuscript accordingly.

**Comment 5:** I agree the authors used the dataset properly as compared to other studies, but here I have a concern about the data. Yes, MCQ180a does give a binomial variable, but MCQ180b gives the age. If the event and time are reporting are large, then there is a possibility that the bio-parameters are not comparable. I suggest perform a sub-analysis to determine if there is an effect.

**Response 5:**

Thank you for your valuable feedback. We fully understand that the event time provided by MCQ180B may impact the comparison between heart failure and biochemical indices. However, upon further examination of the NHANES database, we found that there is a significant amount of missing data regarding the event time in MCQ180B. As a result, we were unable to perform subgroup analysis based on this variable.

We have addressed this issue in the revised manuscript and explained the rationale for using the MCQ160B binary variable to avoid potential bias due to missing data. We believe this approach ensures the robustness of the results and the reliability of the conclusions.

**Comment 6:** Thank you for providing the NHANES variable codes. However, I wish the authors to examine “PHAFSTHR” - Total length of food fast, hours. The authors may notice that some of their fasting participants were not in fasting conditions or have fasted over 24 hours. Please confirm that this variable was used.

**Response 6:**

Thank you for your valuable feedback regarding medical laboratory practices. We fully agree with your point that fasting duration may affect biochemical markers. To ensure the accuracy and reliability of the study results, we have incorporated the PHAFSTHR variable into our latest data analysis and implemented a strict selection criteria for fasting duration.

We excluded participants with fasting durations less than 8 hours or greater than 24 hours, resulting in the exclusion of 567 participants. Subsequently, the results from the univariate regression analysis showed that HOMA-IR (OR = 1.017, 95% CI [1.005 - 1.028]), TyG (OR = 1.884, 95% CI [1.489 - 2.384]), and TyG-BMI (OR = 1.006, 95% CI [1.004 - 1.008]) remained significantly associated with heart failure risk. The exclusion of these non-compliant data did not significantly impact the primary results and conclusions of the study. More importantly, the results remained robust after conducting multivariate regression analysis.

Once again, we appreciate your attention to data quality, and we have ensured the reliability of the data through these rigorous selection steps, further strengthening the validity of the study conclusions.

**Comment 7:** Since R was used, please include all package names that were used and for which analysis, especially DCA and RCS. Also, explain more how DCA was used.

**Response 7:**

Thank you for your valuable feedback on the methods section. Following your suggestion, we have listed all the R packages used in the revised manuscript and clarified the corresponding analysis sections for each package, especially those related to DCA (Decision Curve Analysis) and RCS (Restricted Cubic Spline).

Regarding the use of DCA, we have further elaborated on the implementation steps of the DCA model, including the R packages and relevant functions used, as well as how model comparison and performance evaluation were conducted. We believe these additions will help readers better understand the details of our analytical methods.

**Revised text:**

To examine the nonlinear relationship between HOMA-IR, TyG, TyG-BMI and HF prevalence, restricted cubic spline (RCS) regression analysis was performed, with knots placed at the 10th, 50th, and 90th percentiles. Stratified analyses were conducted based on age, sex, race/ethnicity, diabetes status, and hypertension. Interaction P-values were calculated using likelihood ratio tests in multivariable logistic regression (LRT) models to assess the consistency of relationships across subgroups. A predictive nomogram model incorporating key HF-related variables was developed using multivariable logistic regression, and its discriminative ability was evaluated using receiver operating characteristic (ROC) curves with the area under the curve (AUC) calculation and decision curve analysis (DCA) to assess clinical utility.

To ensure the accurate representativeness of the NHANES 2011-2016 data, we have adjusted the 2-year weight (WTMEC2YR) by dividing it by 3, as per the NHANES guidelines (WTMEC6YR).Missing covariates were handled using the “multiple imputation” method to avoid bias from excluding participants with incomplete data[30,31]. All statistical analyses were performed using R software (version 4.1.6, R Foundation for Statistical Computing, Vienna, Austria), and a P-value < 0.05 was considered statistically significant. The main R packages used in this study included: dplyr (version 1.1.4) for data manipulation and cleaning, ggplot2(version 3.5.1) for data visualization, mice (version 3.16.0) for multiple imputation of missing data, rms (version 6.8-2) for constructing nomogram models, pROC (version 1.18.5) for ROC curve analysis, and car (version 3.1-3) for multivariable regression analysis. These R packages and versions ensure the transparency and reproducibility of the analysis.

**3) Results**

**Comment 8:** Please verify that all percentages are based on the weighted sample size? Lines 155-156, it is usually to give the un-weighted value followed by the weighted percentage. Also, check the percentage of Non-hispanic white = 48.60% (line 157), this number is different in the table.

**Response 8:**

Thank you for your detailed comments regarding the statistical reporting standards. We have reviewed all percentages in the manuscript and confirmed that they are based on weighted sample sizes. Following your suggestion, we have adjusted the presentation by first providing the unweighted sample size, with the weighted percentage indicated in parentheses.

**Revised text:**

The average age of the study population was 48.25 years. A total of 3,727 participants were male (48.36% of the weighted population). Non-Hispanic Whites represented the largest racial group, comprising 2,964 individuals (66.50% of the weighted population). A total of 284 participants had heart failure, corresponding to a weighted prevalence of 3.01%.

**Comment 9:** Line 159: please check the frequency of DM.

**Response 9:**

Thank you for pointing out the error regarding the diabetes frequency. We have identified an issue with our statistical approach for the binary variables. We have reanalyzed and corrected all relevant data, including the frequencies of diabetes, hypertension, as well as race and age. We greatly appreciate the reviewer's professionalism and thoroughness, which has helped enhance the accuracy of our study.

**Revised text:**

Participants with a history of HF were generally older (66.16 years vs. 47.70 years, P < 0.001) and had higher prevalences of diabetes (36.29% vs. 11.93%, P < 0.001) and hypertension (87.32% vs. 48.08%, P < 0.001).

**Comment 10:** Line 160: I am not sure the authors can use the term “baseline”, this is not a longitudinal study.

**Response 10:**

Thank you for pointing out the inaccurate use of terminology. We agree that the term "baseline" is typically used in prospective studies and is not appropriate for the cross-sectional design of our study. Therefore, we have revised the phrase "at baseline" in line 160 to a more accurate description that reflects the nature of the cross-sectional study.

**Revised text:**

Participants in the HF group exhibited higher levels of potassium (P < 0.001), uric acid (P < 0.001), creatinine (P < 0.001), and albumin (P < 0.001) compared with those without HF. Interestingly, participants in the HF group had relatively lower lipid levels, including total cholesterol (P < 0.001), LDL-C (P < 0.001), and HDL-C (P < 0.001). Detailed demographic and clinical characteristics are presented in Table 1.

**Comment 11:** I do not like the format of Table 3. Yes, there are many models in which the authors are showing the change with the addition of the confounding variables as they present 6 outcomes (un-adjusted, 1,2,3,4,5). However, the order of how the covariates are added could change the perspective. I think the reader would be only considered with 3 examples (un-adjusted, 1,5).

**Response 11:**

Thank you for your suggestion regarding the table format. We fully understand that simplifying the models to three (unadjusted, Model 1, and Model 5) could improve the reading experience. However, we have retained the six-step adjusted models to demonstrate the impact of the inclusion of covariates on the regression coefficients, which enhances the transparency and rigor of the model interpretation.

To improve the readability of the table, we have optimized the formatting and provided a detailed description of the covariates adjusted for in each model in the table legend, so that readers can better understand the analytical approach.

**Revised text:**

Data are presented as odds ratios (OR) with 95% confidence intervals (CI). Model 1: Adjusted for gender, age, and race/ethnicity. Model 2: Further adjusted for hypertension, total cholesterol, low-density lipoprotein cholesterol (LDL-C), and serum sodium. Model 3: Additionally adjusted for diabetes. Model 4: Further included potassium, uric acid, alanine aminotransferase (ALT), aspartate aminotransferase (AST), creatinine, and albumin. Model 5: Adjusted for all covariates listed above.

**Comment 12:** For section 3.3, please indicate the evidence at the beginning of the section. The results seemed to be discussed in the results section, please correct this.

**Response 12:**

Thank you for your valuable feedback on the structure of our study. Following your suggestion, we have clarified the sources of evidence at the beginning of Section 3.3, so that readers can better understand the foundational data of the study. Additionally, to avoid mixing the results and discussion, we have separated these sections and made corresponding revisions to the content. We appreciate the reviewer’s attention to the structure of the manuscript, and we believe these changes will further enhance the clarity of the paper.

**Revised text:**

This study evaluated the relationship between HOMA-IR and the TyG index with HF risk across various population characteristics, using data from the NHANES 2011-2016. The analysis revealed that factors such as sex, race, age, diabetes, and hypertension were significantly associated with HOMA-IR, TyG, and TyG-BMI index.

**Comment 13:** Lines 241-248: please include the interaction results with the covariable that is assessed.

**Response 13:**

Thank you for your suggestion regarding the analysis section. We have added the results of the interaction terms and covariate analyses to provide more comprehensive statistical information. Specifically, we have included the relevant tests for the interaction terms and clarified the impact of each covariate on the model. We believe this addition enhances the rigor of the paper and strengthens the reliability of the research conclusions.

**Revised text:**

In exploring the association between the TyG index and HF risk, we found significant heterogeneity across different population characteristics. For females in the Q4 group, the odds ratio was 3.674 (95% CI: 1.986–6.795, P < 0.001), while for males it was 3.499 (95% CI: 1.728–7.082, P < 0.001), indicating that both genders face elevated HF risk with increased TyG index levels. Racial analysis showed that non-Hispanic whites had an OR of 3.728 (95% CI: 1.983–7.010, P < 0.001) in the Q4 group, whereas non-Hispanic blacks exhibited an even higher OR of 4.101 (95% CI: 2.321–7.248, P < 0.001), suggesting heightened susceptibility to HF risk in these populations. Age analysis revealed that the OR for individuals aged ≥60 years in the Q4 group was 2.297 (95% CI: 1.381–3.823), while those aged <60 years reached an OR of 4.116 (95% CI: 1.910–8.874), indicating significant risk among younger individuals. Additionally, the study showed that the TyG index in non-diabetic individuals had an OR of 1.585 (95% CI: 1.168–2.151, P < 0.01), and in the Q4 group, the OR reached 2.863 (95% CI: 1.634–5.018, P < 0.001). The interaction suggests that TyG may be more sensitive in non-diabetic patients (P < 0.05). For hypertension, hypertensive patients in the Q4 group had an OR of 2.750 (95% CI: 1.768–4.277, P < 0.0001), while the association was weaker for individuals without hypertension (P > 0.05). The overall predictive ability of TyG-BMI was similar to that of TyG; however, in diabetic individuals where TyG showed poorer predictive ability, TyG-BMI demonstrated potential, with an OR of 1.003 (95% CI: 1.000–1.005, P < 0.05).

**Comment 14:** Lines 260-264: Please explain the results more clearly.

**Response 14:**

Thank you for your suggestion regarding the presentation of the results. We have revised the relevant content to ensure the results are clearer and easier to understand. Specifically, we have optimized the phrasing to reduce ambiguity and more clearly present the key points of the analysis results. We believe this revision will better assist readers in understanding our research findings.

**Revised text:**

In this study, we systematically assessed the effectiveness of HOMA-IR, TyG, and TyG-BMI in predicting HF risk. Through ROC curve analysis, we found that the area under the curve (AUC) for TyG-BMI (AUC=0.62) was higher than that for TyG (AUC=0.61), which was higher than HOMA-IR (AUC=0.60), indicating the superiority of TyG-BMI and TyG in identifying HF risk (Fig 2). Additionally, decision curve analysis (DCA) further confirmed the clinical applicability of TyG and TyG-BMI across different decision thresholds, demonstrating their higher decision value (Fig 3). In the restricted cubic spline (RCS) curve analysis, both TyG (Fig 4) and TyG-BMI (Fig 5) exhibited a positive correlation trend with HF risk, while HOMA-IR did not demonstrate the same prognostic capability (Fig 6).

**4) Discussion
Comment 15:** Lines 271-275: this being the first paragraph of the discussion should indicate the main results of the study. It does not. Lines 276-297: this information, through interesting, does not discuss the results. This is introduction and only should be included here if the authors attached their results.

**Response 15:**

Thank you for your valuable feedback on the structure of the discussion section. Following your suggestion, we have added an overview of the main findings at the beginning of the discussion to provide readers with a clear summary of the study. Additionally, we have rearranged and adjusted the relevant content, integrating it with the interpretation of the results to ensure that the discussion is more closely aligned with the research findings, rather than being limited to introductory material. We believe these revisions will effectively enhance the logical flow and coherence of the paper.

**Revised text:**

This study explored the effectiveness of HOMA-IR, TyG, and TyG-BMI in predicting HF risk, revealing significant differences in their associations with HF across various population characteristics. Our analysis highlights the potential advantages of TyG in HF risk assessment, while also emphasizing the relevance of HOMA-IR and TyG-BMI in specific subgroups.

**Comment 16:** Lines 301-306: Do not include your results here. We see them above. This section is to discuss them, which is done in lines 306-317

**Response 16:**

Thank you for your comments on the discussion section. Following your suggestion, we have revised lines 301-306 to avoid repeating the research results and have restructured this section to make it more discussion-oriented. For lines 306-317, we have retained the original structure and further refined the interpretation and discussion of the results. We believe these adjustments better align the discussion with its logical structure and effectively enhance the academic quality of the paper.

**Comment 17:** Lines 321-322: How does the “different decision thresholds” highlight the potential risk predictor? Figure 4 is barely mentioned in the results.

**Response 17:**

Thank you for your valuable comments on the results section. Following your suggestion, we have further clarified how different decision thresholds highlight the potential for risk prediction. We have provided a detailed description of the relevant data and analysis results in the results section, and explained the impact of threshold variations on predictive performance. We believe this addition more thoroughly demonstrates the depth of the study and the validity of the conclusions.

**Revised text:**

We further assessed the predictive sensitivity and specificity of HOMA-IR, TyG, and TyG-BMI using ROC curves, DCA curves, and RCS analysis. The AUCs showed that TyG and TyG-BMI have advantages in risk identification. DCA curve analysis also supports the clinical applicability of the TyG index at different decision thresholds, highlighting its potential as a risk prediction tool. TyG-BMI also demonstrated similar advantages. In subgroup analyses by gender and age, the associations between TyG index and TyG-BMI with HF risk showed different predictive abilities across populations. This suggests that clinical risk assessment strategies need to be adjusted based on different population characteristics.

**Comment 18:** Lined 352-353: I cannot agree. Which evidence and test confirms this statement.

**Response 18:**

Thank you for your feedback on the conclusion section. Following your suggestion, we have revised and improved the conclusion. We have added more solid evidence and analytical results to better support our conclusions. These additional analyses not only enhance the reliability of the conclusions but also provide a stronger scientific basis. We believe this revision more clearly articulates the core findings of the study.

**Revised text:**

This study evaluated the effectiveness of HOMA-IR, TyG, and TyG-BMI in predicting HF risk. The results indicate that TyG has a stronger association with HF risk than HOMA-IR and TyG-BMI, and it outperforms HOMA-IR in predicting HF risk, especially in specific subgroups. Our findings highlight the potential of the TyG index as a tool for HF risk assessment, warranting further exploration of its mechanisms and clinical applications to improve early identification and management of HF.

**Reviewer #4: (No Response)**
